# A one‐stop shop model for improved efficiency of pre‐exposure prophylaxis delivery in public clinics in western Kenya: a mixed methods implementation science study

**DOI:** 10.1002/jia2.25845

**Published:** 2021-12-12

**Authors:** Stephanie D. Roche, Josephine Odoyo, Elizabeth Irungu, Benn Kwach, Annabell Dollah, Bernard Nyerere, Sue Peacock, Jennifer F. Morton, Gabrielle O'Malley, Elizabeth A. Bukusi, Jared M. Baeten, Kenneth K. Mugwanya

**Affiliations:** ^1^ Department of Global Health University of Washington Seattle Washington USA; ^2^ Centre for Microbiology Research Kenya Medical Research Institute Nairobi Kenya; ^3^ Partners in Health and Research Development Nairobi Kenya; ^4^ Department of Obstetrics and Gynecology University of Washington Seattle Washington USA; ^5^ Department of Medicine, University of Washington Seattle Washington USA; ^6^ Department of Epidemiology, University of Washington Washington Seattle USA; ^7^ Gilead Sciences Foster City California USA

**Keywords:** HIV prevention, implementation science, Kenya, organizational efficiency, pre‐exposure prophylaxis, quality improvement

## Abstract

**Introduction:**

In public clinics in Kenya, separate, sequential delivery of the component services of pre‐exposure prophylaxis (PrEP) (e.g. HIV testing, counselling, and dispensing) creates long wait times that hinder clients’ ability and desire to access and continue PrEP. We conducted a mixed methods study in four public clinics in western Kenya to identify strategies for operationalizing a one‐stop shop (OSS) model and evaluate whether this model could improve client wait time and care acceptability among clients and providers without negatively impacting uptake or continuation.

**Methods:**

From January 2020 through November 2020, we collected and analysed 47 time‐and‐motion observations using Mann–Whitney U tests, 29 provider and client interviews, 68 technical assistance reports, and clinic flow maps from intervention clinics. We used controlled interrupted time series (cITS) to compare trends in PrEP initiation and on‐time returns from a 12‐month pre‐intervention period (January–December 2019) to an 8‐month post‐period (January–November 2020, excluding a 3‐month COVID‐19 wash‐out period) at intervention and control clinics.

**Results:**

From the pre‐ to post‐period, median client wait time at intervention clinics dropped significantly from 31 to 6 minutes (*p* = 0.02), while median provider contact time remained around 23 minutes (*p* = 0.4). Intervention clinics achieved efficiency gains by moving PrEP delivery to lower volume departments, moving steps closer together (e.g. relocating supplies; cross‐training and task‐shifting), and differentiating clients based on the subset of services needed. Clients and providers found the OSS model highly acceptable and additionally identified increased privacy, reduced stigma, and higher quality client–provider interactions as benefits of the model. From the pre‐ to post‐period, average monthly initiations at intervention and control clinics increased by 6 and 2.3, respectively, and percent of expected follow‐up visits occurring on time decreased by 18% and 26%, respectively; cITS analysis of PrEP initiations (*n* = 1227) and follow‐up visits (*n* = 2696) revealed no significant difference between intervention and control clinics in terms of trends in PrEP initiation and on‐time returns (all *p*>0.05).

**Conclusions:**

An OSS model significantly improved client wait time and care acceptability without negatively impacting initiations or continuations, thus highlighting opportunities to improve the efficiency of PrEP delivery efficiency and client‐centredness.

## 1 INTRODUCTION

In the 6 years since the World Health Organization released guidelines recommending daily oral tenofovir‐based pre‐exposure prophylaxis (PrEP) for populations at substantial risk of HIV infection [1], 21 countries in sub‐Saharan Africa (SSA) have implemented pilot or national PrEP programs [2]. From 2016 to 2020, these programs initiated 500,000 individuals on PrEP [2]; however, epidemiologists estimate the current rate of PrEP uptake in SSA is still too slow to significantly impact population‐level HIV incidence [3]. Drawing on lessons learned from differentiated service delivery (DSD) for antiretroviral treatment (ART) [4], recent PrEP implementation efforts in SSA and globally have focused heavily on diversifying PrEP models in terms of delivery location, provider type, frequency of contact, and package of services offered [5, 6]. Many DSD models have moved PrEP delivery outside of clinical settings [4, 6] to circumvent barriers, such as long wait times, HIV stigma, insufficient privacy, and poor treatment from healthcare providers [7, 8, 9, 10, 11]. Less attention, however, has been given to improving existing models of PrEP delivery used in public healthcare facilities. Recognizing that such facilities will play a key role in achieving PrEP at scale, some PrEP implementers have called for “PrEP delivery optimization” [12, 13, 14, 15], including the Kenya Ministry of Health (MOH), which encouraged the development of “facility‐based fast track models” [16, p. 61] in its latest strategic AIDS framework.

Currently, in most public healthcare facilities, services are delivered sequentially by different providers at different delivery points (e.g. HIV testing services [HTS] delivered by HTS providers at HTS points; dispensing done by pharmaceutical technologists at the pharmacy) with clients moving between, and frequently queueing at, each. One promising option for optimizing PrEP delivery is a “one‐stop shop” (OSS), a broad term for models that deliver all necessary services at a single touchpoint. Whereas some co‐locate services so clients receive them “under the same roof,” others further reorganize services so clients receive them in a single room and/or from a single provider [17, 18]. Regardless of OSS configuration, the underlying rationale is the same: to increase care acceptability among clients and improve efficiency by eliminating unnecessary wastes, such as queueing and movement. In SSA, OSS models have primarily been used to integrate previously separate service lines, like HIV and TB [19, 20, 21, 22] or family planning (FP) services [23, 24, 25, 26, 27, 28]. An OSS model that consolidates the core components of the PrEP intervention (HTS, counselling, clinical assessment, and drug dispensing) has yet to be implemented and evaluated within the context of routine PrEP delivery in SSA. As countries scale up PrEP, they may benefit from understanding how some, or all, of these steps might be consolidated and the impact on client care experiences. We, therefore, conducted a mixed methods study at four public healthcare facilities in Kenya to identify strategies for operationalizing an OSS model and assess whether this model could improve efficiency and care acceptability among clients and providers without negatively affecting uptake and continuation.

## 2 METHODS

### 2.1 Study background and design

Beginning in 2017, the Partners Scale‐Up Project (PSUP) catalyzed national scale‐up of PrEP through provision of technical assistance (TA), initially to 25 clinics and later to over 100 clinics [29]. In August 2019, PSUP presented the OSS concept to MOH and clinical leaders of 12 PrEP clinics in Western province. Clinics interested in piloting an OSS submitted to PSUP a plan detailing how they would operationalize their OSS model (e.g. where they would set it up and which cadre of providers they would involve). From September to December 2019, PSUP facilitated meetings at prospective study sites to gather feedback from comprehensive care clinic (CCC), HTS, and pharmacy providers, which clinics subsequently used to refine their OSS proposals. PSUP also administered to staff a validated survey [30] to assess organizational readiness for change (ORIC). Ultimately, PSUP selected four clinics based on proposal strength, staff support for piloting an OSS, and ORIC scores. Like most PrEP facilities in Kenya, the four sites selected were subcounty hospitals that delivered PrEP in their HIV CCCs using national PrEP guidelines. The surrounding area of clinics A and B was peri‐urban, whereas that of clinics C and D was rural.

For this pilot, clinics A and B established their OSS in their differentiated care clinics (where stable clients receive express ART services) and discontinued PrEP delivery in their CCCs. Clinics C and D established their OSS in their FP clinic and gender‐based violence (GBV) clinic, respectively, and in addition, continued to offer regular (non‐OSS) PrEP services at their CCCs. Clinics made their OSS models fit for purpose. In all clinics’ baseline OSS models, the following services and delivery‐related tasks were slated to occur within a single room: client file retrieval, clinical review, adherence counselling, prescription writing, and dispensing. Clinic B and D's baseline models additionally featured in‐room vital signs assessment. Lastly, whereas clinics A, B and C planned for OSS clients to obtain HIV testing at an HTS point located in the same building as the OSS, clinic D's baseline model featured in‐room HIV testing. Table S1 of the appendix further details clinics’ PrEP delivery models pre‐ and post‐intervention.

Our study used a convergent mixed methods design [31], with quantitative and qualitative data collected simultaneously and given equal weight during analysis [32]. Our primary outcomes of interest were (1) implementation strategies (i.e., actions taken to implement the OSS); (2) implementation challenges; (3) client wait time (time spent waiting or walking to receive PrEP services), which we hypothesized would decrease from the pre‐ to post‐intervention period, and (4) acceptability of PrEP services, which we hypothesized would improve under the OSS model. Although the OSS intervention was not intended to change time spent with a provider (contact time), PrEP initiations, or PrEP continuations, we assessed these as secondary outcomes. For our analysis of PrEP initiations and continuations, we selected four clinics — two peri‐urban and two rural — to serve as control clinics. These clinics had similar pre‐intervention levels and trends in PrEP initiations and continuations as the four OSS clinics. We originally planned to launch the OSS in January 2020 and have a 12‐month pre‐intervention period (January–December 2019) and a 4‐month post‐intervention period (January–April 2020). Ultimately, we adjusted these accordingly for clinics C and D, which launched their OSS in February 2020, and to mitigate the influence of the COVID‐19 pandemic, we expanded all clinics’ post‐intervention periods to November or December 2020 (depending on OSS launch date) and treated April through June 2020 as a wash‐out period. This study was approved by the institutional review boards of the University of Washington (STUDY00002183) and the Kenya Medical Research Institute (P00040/3338), which did not require individual consent for client data collected as part of routine health services; written informed consent was obtained for interviews.

### 2.2 Data collection

#### 2.2.1 Quantitative

PSUP staff abstracted data on demographics (e.g., sex, age, and martial status), HIV risk factors and PrEP use (e.g., PrEP initiate date and refill history) from clinical records of individuals who initiated PrEP and/or received follow‐up care from January 2019 through November 2020 and entered it into SurveyCTO (Dobility, Inc., Cambridge, MA, USA). At study baseline and endline, trained PSUP staff used a structured tool to conduct 47 time‐and‐motion observations of randomly selected PrEP initiation and follow‐up visits at OSS clinics on different days of the week and times of day. Total service time was divided into contact time and wait time.

#### 2.2.2 Qualitative

During the post‐period, technical assistants conducted bimonthly clinic visits and generated clinic flow maps and reports (*n* = 69) on implementation strategies used and challenges encountered, model modifications, and OSS acceptability. In October and November 2020, Kenyan qualitative researchers (authors BK and AD) conducted interviews with (1) healthcare providers employed by an OSS clinic or the County Department of Health and (2) PrEP clients. Eligible individuals delivered or oversaw the delivery of OSS PrEP services (provider group) or obtained PrEP at an OSS at least once (client group), were age 18 or above, and self‐reported comfort communicating in English. We anticipated that 12 client interviews (three per clinic) and 12 provider interviews (three per clinic) would be sufficient to answer our qualitative research questions, which were narrow in scope (e.g., to understand whether participants liked delivering/receiving PrEP services at the OSS). We used purposive sampling to recruit providers of different cadres and clients of different sexes, ages and exposure (yes/no) to the clinic's pre‐OSS model. We developed semi‐structured interview guides that solicited, from providers, details about OSS operations, barriers and facilitators, and perceived advantages and disadvantages, and, from clients, OSS visit descriptions, perceptions of care quality and recommendations for improvement. All interviews were conducted one‐on‐one, in English, in a private room or via phone, audio recorded and transcribed verbatim by the interviewer, with transcripts spot‐checked for quality by author SDR.

### 2.3 Data analysis

#### 2.3.1 Quantitative

Our primary outcome was client wait time, as captured in time‐and‐motion observations. We performed Mann–Whitney U tests to assess whether median wait time differed significantly from the pre‐ to post‐periods. Secondary outcomes included clinic‐level rates of PrEP initiation and percent of expected follow‐up visits that occurred on time, with “on time” defined as “occurring within two weeks of the date the client would run out of PrEP pills, according to dispensing records.” We assessed descriptive statistics of clients and collapsed data to obtain monthly counts of our outcomes. We compared OSS and control clinics using a controlled interrupted time series (cITS) approach. We modelled incidence rate ratios with negative binomial models with first‐order autoregressive structure and included random intercepts and random slopes to account for clustering by clinic and clinic‐level heterogeneity in intercepts and trends over time. Each model included fixed effects for study month, treatment group (intervention vs. control), number of months since OSS implementation and interactions for each pairwise combination to allow estimation of the pre‐ and post‐implementation time trends and the immediate effect of implementation on the outcomes of interest. All quantitative analyses were conducted using RStudio (RStudio Team, version 1.4.999).

#### 2.3.2 Qualitative

Interviews and TA reports were analysed using a combination of directed and conventional content analysis [33]. Our codebook included deductive codes for the types of waste in Ohno's model for continuous quality improvement, implementation strategies from a modified version of the Expert Recommendations for Implementing Change [34, 35] and change concepts identified by Langley et al. [36, 37], as well as inductive codes identified during repeated readings of the data [38]. Author SDR drafted interview memos with a summary of key points for each code, quotations and analytic reflections [39] that drew comparisons across participants and datasets and synthesized findings into higher level themes [40]. Memos were reviewed by the interviewers, with disagreements resolved through group discussion. Simultaneous integration [41] of qualitative and quantitative data was further achieved through the development of joint displays to determine common concepts and explore how results confirmed, contradicted, or expanded upon one another [42]. To highlight the relationship between actions and improvement, we organized the identified implementation strategies according to change concepts compiled by Langley et al. [37]. We also categorized the specific implementation challenges clinics encountered according to the Tailored Implementation in Chronic Disease (TICD) checklist [43, 44]. Table S2 of the appendix contains additional details on our methodology.

## 3 RESULTS

### 3.1 Participants and data

#### 3.1.1 PrEP clients

During the 12‐month pre‐intervention period, intervention and control clinics initiated 385 and 212 clients on PrEP, respectively. During the 8‐month post‐intervention period, intervention and control clinics initiated 410 and 220 clients on PrEP, respectively. In both groups during both periods, approximately 60% of clients were female and about 75% were 18‐ to 34‐years‐old and in a known serodiscordant relationship (Table 1). Intervention and control clinics had, respectively, 1276 and 620 follow‐up visits during the pre‐intervention period and 523 and 277 follow‐up visits during the post‐intervention period. Distributions of sex, age, and HIV risk factors were similar across groups and periods.

**Table 1:** Demographic characteristics of (A) clients who initiated PrEP and (B) clients who received follow‐up PrEP care during the study

		Pre‐intervention period^a^		Post‐intervention period^b^	
Analysis	Characteristic	Intervention (*N* = 385)	Control (*N* = 212)	Intervention (*N* = 410)	Control (*N* = 220)
(A) PrEP initiations	Female sex – no. (%)	237 (62)	118 (56)	249 (61)	157 (71)
	Age – no. (%)				
	18–24	128 (33)	52 (25)	115 (28)	80 (36)
	25–34	156 (41)	106 (50)	197 (48)	76 (35)
	35–44	62 (16)	29 (14)	70 (17)	41 (19)
	≥ 45	39 (10)	25 (12)	28 (7)	23 (10)
	Married or cohabitating	342 (89)	171 (81)	340 (83)	129 (59)
	HIV risk factors – no (%)				
	Sex partner(s) HIV+	285 (74)	150 (71)	281 (69)	127 (58)
	Sex partner(s) high risk and HIV status unknown	90 (23)	47 (22)	119 (29)	50 (23)
	Multiple sex partners and no consistent condom use	45 (12)	13 (6)	28 (7)	13 (6)
		Intervention (*N* = 389)	Control (*N* = 241)	Intervention (*N* = 332)	Control (*N* = 155)
(B) PrEP continuation	Follow‐up visits – no. (%)				
	1	126 (32)	79 (33)	204 (61)	71 (46)
	2	75 (19)	63 (26)	88 (27)	53 (34)
	≥ 3	188 (48)	99 (41)	40 (12)	31 (20)
	Female sex – no. (%)	237 (61)	149 (62)	199 (60)	97 (63)
	Age – no. (%)				
	18–24	77 (20)	42 (17)	68 (20)	34 (22)
	25–34	176 (45)	119 (49)	152 (46)	72 (46)
	35–44	81 (21)	47 (20)	73 (22)	30 (19)
	≥ 45	55 (14)	33 (14)	39 (12)	19 (12)
	Married or cohabitating	374 (96)	218 (90)	306 (92)	111 (72)
	HIV risk factors – no (%)				
	Sex partner(s) HIV+	339 (87)	197 (82)	275 (83)	109 (70)
	Sex partner(s) high risk and HIV status unknown	56 (14)	47 (20)	60 (18)	30 (19)
	Multiple sex partners and no consistent condom use	31 (8)	8 (3)	27 (8)	15 (10)

^a^ Pre‐intervention period: 1 January 2019–31 December 2019 for all sites except two in the intervention group whose pre‐intervention period end date is 14 February 2020.

^b^ Post‐intervention period: 1 January 2020–30 November 2020 (excluding wash‐out period of 1 April 2020–30 June 2020) for all sites except two in the intervention group whose post‐intervention period start date is 15 February 2020.

#### 3.1.2 Interview participants

We interviewed 14 providers and 15 clients. Providers included six clinical officers, four nurses, two counsellors, and two administrators; 43% (6/14) were female, and median age was 34 (interquartile range [IQR]: 33–38). The client sample was 53% female (8/15) and had a median age of 39 (IQR 29–41). Most clients (13/15) were married, and all had children. About half (7/15) experienced PrEP delivery under both the pre‐OSS and OSS models. Additional demographic details are available in the appendix (Table S3).

### 3.2 OSS impact on PrEP initiations and on‐time returns

From the pre‐ to the post‐period, the average monthly number of PrEP initiations increased by 6 at OSS clinics (from 7.6 to 13.6) and by 2.3 at control clinics (from 4.5 to 6.8); results of cITS analyses revealed no significant difference between OSS and control clinics with respect to immediate change in initiations at the time of OSS implementation (*p* = 0.5) or over time (*p* = 0.4). From the pre‐ to the post‐period, the average monthly percent of expected follow‐up visits that occurred on time decreased by 18% at OSS clinics (from 70% to 52%) and by 26% at control clinics (from 70% to 42%); cITS analyses again found no significant difference between OSS and control clinics with respect to immediate change (*p* = 0.08) in this outcome or change over time (*p* = 0.6).

### 3.3 OSS impact on service time

**Figure 1:**
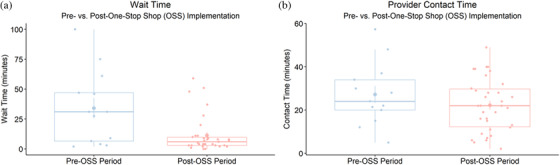
Client wait time and provider contact time. Boxplots of client wait time (panel a) and provider contact time (panel b) at intervention clinics before and after implementing the one‐stop shop. Abbreviation: OSS, one‐stop shop.

Median client wait time dropped significantly from the pre‐ to post‐period (31 minutes [IQR 0–71] vs. 6 minutes [IQR 0–13], *p* = 0.02), but median provider contact time remained the same (24 minutes [IQR 10–38] vs. 22 minutes [IQR 5–40], *p* = 0.38) (Figure 1). Analysis of clinic flow maps, TA reports and interviews suggests that clinics achieved this efficiency gain by operationalizing three main change concepts via five discrete implementation strategies (highlighted in bold below and detailed in Table 2).

**Table 2:** Strategies used by sites to implement the one‐stop shop (OSS) and illustrative quotes on how changes affected delivery inefficiencies (wastes)

Change concept^a^ Specific strategy used^b^	Effect on client/provider^c^	Waste reduced (for whom)	Illustrative quotes
Redirect away from bottlenecks Change service sites: moved PrEP delivery to a lower volume department.	Reduced time to service start, as the queue to see the PrEP provider at the OSS was generally shorter compared to previous delivery location.	Waiting (client)	**Lower client volume** : “The waiting time has reduced because, at this other side [the OSS], they are not as many [clients] as in the HIV clinic [CCC].” *(Female hospital administrator, Site C)*
Move steps closer together Redesign workflow: relocated client files, equipment, and drugs to OSS. Revise professional roles and conduct ongoing training: cross‐trained and task‐shifted so other cadres could complete parts of PrEP delivery (e.g., dispensing).	(B and C) Enabled client record retrieval, vitals assessment, and drug dispensing to take place in the OSS, thus eliminating client movement to (and potential queueing at) the records department, triage area, and pharmacy. (B) Reduced provider movement to obtain client files and PrEP register. (B) Enabled in‐flow documentation, which improved accuracy and reduced time spent reconciling discrepancies.	Motion (client) Waiting (client) Motion (provider) Defects (provider)	**Less movement, fewer queues, fewer providers** : “Everything is done under one roof [at the OSS]. I get weighed, counseled, and just get PrEP from the same room, unlike [before at the CCC] … where I had queue in each and every point I went to because the providers were [busy] attending to other clients. … Today, I only saw one clinician. Before, there were many and, therefore, there was no privacy.” *(Female PrEP client, Site C)* **Less provider movement** : “All the commodities are within the [OSS] room, be it the [appointment] diary, the PrEP files—everything. When [PrEP] clients come in, the [OSS] provider just picks the client's file and does everything within the room, unlike [before at] the CCC, [where] when they get a [PrEP client] in a different room, he or she is forced to stand up and go to the room where things are being kept and take everything to the room, then to the [data] clerk, then to the client, etc. So there is less movement now.” *(Female nurse, Site C)* **In‐flow documentation** : “[Previously,] we had different providers giving PrEP the same week. So if there was a problem in the documentation, it was difficult to resolve. … [Now] we have only one person [delivering PrEP] who is answerable to anything.” *(Male clinical officer, Site B)*
Use differentiation^d^ Obtain and use client feedback:^e^ designated a clinician to attend to OSS clients and fast‐tracked PrEP clients to him/her Revise professional roles: task‐shifted so lower level cadre could attend to refill‐only clients	(D) Reduced client waiting by allowing OSS clients to bypass other clients in the clinic (e.g. ART clients and FP clients). (E) Reduced client waiting by allowing refill‐only clients to be seen by a lower level cadre of provider, as opposed to making them wait for the OSS clinician to become available.	Waiting (client)	**Task‐shifting** : “Currently, I can attend to the clients coming for refills … so as to reduce the time it would take for the clinician to come.” (*Male peer educator, Site A*)

Abbreviations: ART, antiretroviral therapy; CCC, comprehensive care clinic; OSS, one‐stop shop; PrEP, pre‐exposure prophylaxis.

^a^ From Langley et al.’s compilation of change concepts.

^b^ From Perry et al.’s modified version of the Expert Recommendations for Implementing Change (ERIC) framework.

^c^ As reported by clients and providers in interviews and TA visits.

^d^ At two sites, the designated OSS clinician attends to PrEP clients as soon as s/he was next available. A third site implemented fast‐tracking at the HTS point only.

^e^ In the years leading up to OSS implementation, clinics had received feedback from PrEP clients, especially those only coming for refills (i.e., not due for their quarterly HIV testing and clinical review), that they were dissatisfied with the single queue for ART and PrEP clients. Clinics had previously attempted to fast‐track PrEP clients in the CCC, but fast‐tracking was not implemented reliably prior to the establishment of the OSS.

#### 3.3.1 Change concept 1: redirect away from bottlenecks

Although a few providers noted occasional back‐ups at the OSS, all providers agreed that, on average, moving PrEP delivery to a lower volume department (**Change Service Sites**) reduced client wait time:

[At the CCC] we have both those who are on [ART] care and those who are coming for PrEP. … But here [at the OSS], we are only dealing with Gender‐Based Violence [clients], which are less. So they [PrEP clients] spend shorter period here before they are attended to. *(Male clinical officer, clinic D)*

One provider felt that the OSS especially benefits PrEP follow‐up clients who require little time with a provider:

[Imagine] someone has just come for a [PrEP] refill. You know, [for a] refill, you just come, take your drugs, do a quick follow‐up, and leave. So there's no need of putting these [PrEP] clients waiting for long at the [CCC] queue.” *(Male clinical officer 2, clinic B)*

Clients similarly characterized the CCC as overcrowded and reported that moving PrEP delivery out of the CCC reduced HIV stigma:

The wait time has gone down. Previously, I'd sometimes wait for 2 hours before being seen [at the CCC]. It's because we were mixed together with ART clients, which also wasn't good because some fear someone might look at you and say you're HIV‐positive. *(Female PrEP client, clinic A)*

Providers, especially those new to PrEP delivery, expressed relief that OSS workload was manageable. Some also reported that the OSS reduced CCC providers’ workload:

[CCC providers] are happy that they have been relieved of [PrEP] delivery. They used to handle [ART] duties and then attend to PrEP clients as well. Now they are left with their key [ART] duties. *(Male peer educator, clinic A)*

#### 3.3.2 Change concept 2: move steps closer together

Providers reported that the OSS model consolidated many of the core components of PrEP by relocating the necessary supplies **(Redesign Workflow)**, such as client files, PrEP drugs, and appointment book, to the OSS and shifting some PrEP delivery tasks to other cadres **(Revise Professional Roles; Conduct Ongoing Training)**.

In the CCC, the way it's designed there, the stations are at different points. The reception is at [a] different area, the pharmacy at [a] different area, clinical room at a different place. The One‐Stop Shop is beneficial because you break the issue of … having these [PrEP] clients moving from one point to the other. You just have everything there. *(Male clinical officer 1, clinic B)*

**Figure 2:**
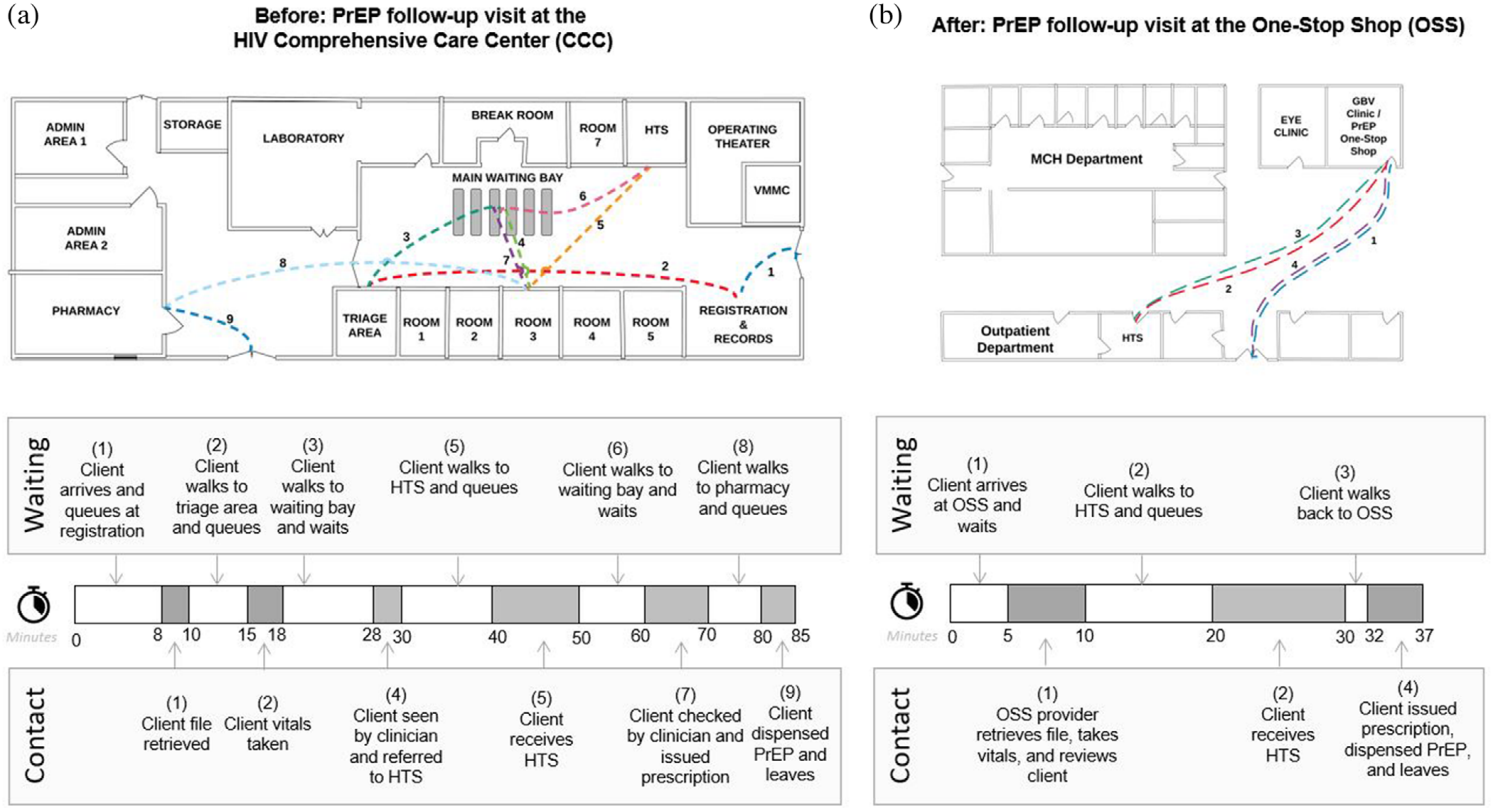
Client movement and service time. Example breakdown of client movement and service time before (panel a) and after (panel b) implementation of the one‐stop shop. Abbreviations: CCC, comprehensive care centre; GBV, gender‐based violence; HTS, HIV testing services; MCH, maternal and child health; OSS, one‐stop shop; PrEP, pre‐exposure prophylaxis; VMMC, voluntary medical male circumcision.

Figure 2 further illustrates how changes to clinic and client flow eliminated stops at the registration, triage and pharmacy areas, thus reducing client movement and queueing.

In addition to the time and motion savings, most clients reported that this consolidation enhanced their privacy by reducing the number of providers they see and enabling them to skip the pharmacy.

[At the OSS] they just give [PrEP] to you, you put it in your bag, and leave. No one will know I am taking PrEP because I don't have to go the pharmacy to queue with everyone else. *(Female PrEP client, clinic C)*

Some providers reported that centrally locating PrEP supplies reduced their movement around the clinic and facilitated more accurate, in‐flow PrEP documentation. Task‐shifting resulted in some providers having more responsibilities (e.g., vital signs assessment and PrEP dispensing) under the OSS model than the pre‐intervention model; yet, when asked how they felt about these additional tasks, providers reported that they did not present a significant burden, were worthwhile because they made clients happier, and, in some cases, made it easier for them to carry out their primary PrEP delivery responsibilities:

Previously, we sent clients to CCC triage for vitals [signs assessment]. But now we have a scale in the OSS, and I just do it there myself in two minutes. Now I'm not waiting for the client to come back [from triage], and it is faster and easier [for the client] than queueing at the triage, so everyone's happy. *(Male clinical officer, clinic A)*

HIV testing was the one component of PrEP delivery not performed within any clinic's OSS. Time‐and‐motion data indicated that travelling to/from an HTS point and queueing there typically accounted for 55% of clients’ wait time (IQR: 39–100%). About half of providers (7/14) and clients (7/15) recommended adding in‐room HIV testing to the OSS.

#### 3.3.3 Change concept 3: use differentiation

In response to PrEP clients’ complaints about being lumped together with ART clients, two clinics differentiated PrEP clients from others by fast‐tracking PrEP clients to the front of a designated OSS clinician's queue **(Obtain and Use Client Feedback)**. A third clinic implemented fast‐tracking at the HTS point. Three clinics additionally differentiated clients requiring clinician attention (e.g., clients initiating PrEP) from those who did not (e.g., follow‐up clients with no issues) by allowing counsellors to fully attend to the latter **(Revise Professional Roles)**.

### 3.4 Implementation challenges

Early challenges (bolded hereafter and detailed in Table 3) included **Schedulin** **g** and **Delegation of Tasks** at clinics A and B, both of which initially designated a single clinician to run the OSS and struggled when s/he was off service or busy with other clients. Clinics addressed this issue by implementing a rotating schedule so the OSS was always staffed and by task‐shifting PrEP refill distribution from clinicians to peer educators. At clinic C (FP clinic), insufficient **Domain Knowledge** about PrEP delivery and a heavy FP **Workload** initially led some to resist delivering PrEP. In response, this clinic provided additional PrEP training and assigned two providers per shift to the OSS. Although clinic D (GBV clinic) originally planned to do in‐room HIV testing, existing **Regulation, Rules, and Policies** made this infeasible, as there was no established pathway for the OSS to order testing kits from the clinic's store, and the HTS Department protested that HIV testing was only allowed in designated HTS points. At clinics A and B, as part of COVID precautions, the HTS point closest to the OSS was closed for most of the post‐OSS period; thus, suboptimal **Resource Proximity** led to clients walking farther to receive HTS. Lastly, 6 months post‐OSS implementation (July 2020), clinic B was designated a COVID isolation centre and had to relocate its OSS to the CCC. A few clients complained that this move failed to meet their **Patient Preferences** to receive PrEP services in a clinic area not associated with HIV care.

**Table 3:** Provider‐reported challenges to implementing the one‐stop shop (OSS) model

TICD determinant^a^	Implementation challenge	Adjustments made
Scheduling Delegation of tasks Workload Domain knowledge	Staffing Initially, only one clinician worked at the OSS, making PrEP delivery difficult when that clinician was on leave or unavailable *(clinics A and B)*	Clinics implemented a rotating schedule whereby different clinicians were assigned to attend to OSS clients on a weekly or monthly basis
	Initially, OSS clinician was stationed at the OSS and responsible for attending only to PrEP clients; but after COVID‐19, s/he was stationed at the CCC and also responsible for ART clients. S/he found it hard to move back and forth between CCC and OSS (*clinics A and B)*	Non‐clinician OSS staff began attending to refill‐only clients and completing majority of steps for new and follow‐up clients on their own, calling the clinician over to the OSS only as needed (e.g., for prescription writing).
	Initial pushback from provider designated to deliver PrEP at OSS who felt unprepared to deliver PrEP and that the workload was too heavy for a single provider *(clinic C)*	Additional PrEP training provided; hospital administration increased the number of providers at OSS to two and implemented a rotating schedule
Regulation, rules, and policies Resource proximity	HIV testing HTS point closest to OSS temporarily closed as part of COVID‐19 precautions *(clinics A and B)* In‐room HIV testing not implemented as planned because no existing system for OSS to order HIV testing kits from clinic store and HTS department opposed HIV testing outside of designated HTS points *(clinic D)*	OSS clients sent to HTS point in other hospital department; OSS providers often tried to attend to other clients in the meantime, though this sometimes led to clients waiting upon return from HTS
Patient preferences	Stigma Due to COVID‐19, OSS relocated to the CCC, where some PrEP clients feel HIV‐related stigma *(clinic B)*	OSS clients are fast‐tracked to a separate room in the corner of the CCC

Abbreviations: ART, antiretroviral therapy; CCC, comprehensive care clinic; HTS, HIV testing services; OSS, one‐stop shop; PITC, provider‐initiated testing and counseling; PrEP, pre‐exposure prophylaxis; TICD, Tailored Implementation in Chronic Diseases checklist.

^a^ Determinants of implementation from Squires et al.’s modified version of the Tailored Implementation in Chronic Diseases (TICD) checklist, originally developed by Flottorp et al. (2013) [43].

### 3.5 Impact on service quality

Whereas some provider interviewees expressed hope that the OSS would eventually increase PrEP initiations and improve continuation, a few viewed the primary value of the OSS as better meeting clients’ care preferences, a benefit also reported by many clients.

I loved the time I had with the [OSS] providers because, before [at the CCC], they never took a lot of time with me to share the important things. … So I am seriously very happy. *(Female PrEP client, clinic B)*

I liked the [OSS] experience. I didn't feel rushed at any point. They even offered me time to ask questions. … Today I was treated way much better as compared to before. … [Back] at CCC, they generally were not friendly. [They would say,] ‘What do you want? Sit there.’ … But a hospital needs a friendly atmosphere where someone will start recovering even before seeing a healthcare provider. *(Male PrEP client, clinic A)*

## 4 DISCUSSION

Delivery inefficiencies threaten to undermine the public health impact of PrEP by tempering both client willingness to access and continue PrEP and provider ability to deliver PrEP services at public health facilities. Although an increasing variety of private sector and/or non‐facility‐based delivery models are being tested in Kenya and other parts of SSA (e.g., PrEP delivery to adolescent girls and young women in community safe spaces [45]; retail pharmacy‐based PrEP delivery [46]), public health facilities are currently the main purveyors of PrEP in SSA and will likely remain so as countries scale PrEP up nationally because of their potential reach. Our study adds to the PrEP delivery science by identifying a basic change package of low‐cost, easy‐to‐implement strategies that enabled public clinics to significantly reduce client wait time and improve care acceptability among clients and providers. The reported benefits of the OSS included not only less waiting time (queueing and movement) but also reduced stigma, enhanced privacy, and higher quality client–provider interactions. Though specific to Kenya, our findings may have broad applicability to other public health systems in SSA that have similarly been organized around delivering curative care through highly differentiated service lines [47].

Throughout SSA, PrEP is being added to public health systems that are already resource‐constrained, resulting in challenges for provider buy‐in [48, 49, 50]. Providers in our study, however, demonstrated their willingness to change how they deliver PrEP, even if this meant taking on additional work. Their emphasis on how the OSS made their clients happy suggests that clinicians were motivated, in part, by positive client feedback. This finding aligns with other qualitative studies with PrEP providers [51, 52], as well as theories from behavioural and implementation science, which posit that provider willingness to adopt an innovation is driven, in part, by feelings of purpose [53] and belief that the innovation will confer a relative advantage [54]. Providers may also have been motivated to change their delivery practices because of the efficiency gains it created for them (e.g., less room‐to‐room movement), which freed up time for them to spend with other clients. Overall, our findings suggest that, even in resource‐constrained settings, providers may be more willing to take on PrEP delivery when the model is efficient and person‐centred. Ensuring that providers understand these benefits will likely be an important step for securing their support.

Task‐shifting is a commonly used strategy for addressing human resource constraints across SSA [55], especially for ART delivery [56, 57]. Similarly, the clinics in our study task‐shifted specialized tasks “down” to lower level cadres, such as moving PrEP dispensing from pharmaceutical technologists to peer educators; however, contrary to prevailing practices, clinics also achieved efficiency gains by moving less specialized tasks “up” to higher level cadres. For example, at times, clinicians, instead of peer educators, took vital signs. Although in many contexts, task‐shifting “up” would be considered a poor use of a rare resource (a clinician), our study found that, in the context of highly fragmented service delivery and unreliable wait times at other service delivery points, task‐shifting “up” simple tasks with short cycle times makes sense as an improvement strategy. This strategy also worked well in this context because clinics relocated all necessary supplies to the OSS room, thereby ensuring that the time OSS providers spent with clients was predominantly “value‐add” and not wasted searching for or retrieving materials from other clinic areas. In short, task‐shifting “up” in this context corrects for some of the negative consequences of an organizational structure that prioritizes differentiation by function over coordination of functions [58]. Our finding that PrEP clients strongly prefer to see fewer providers also aligns with prior studies on ART client care preferences [59, 60, 61]. Future research is needed to investigate the impact of task‐shifting “up” on PrEP clinician productivity and to evaluate the acceptability of alternative OSS models. In light of our finding that over half of client wait time was for travel to/from and queueing at an HTS point, future iterations of the OSS model should test additional interventions, such as HIV self‐testing, that could potentially expedite the HTS component. For example, a recent study at a subcounty hospital in Western province piloted the use of in‐room, oral fluid‐based HIV self‐testing (HIVST) for PrEP continuation and found that clients who opted for HIVST had significantly shorter clinic visits [62]. A randomized controlled trial currently underway in Central province is assessing whether dispensing clients a 6‐month supply of PrEP and allowing them to complete quarterly HIV testing at home via an oral fluid‐ or blood‐based HIVST leads to better adherence and continuation [63].

In African ART programs, DSD models for stable and not‐yet stable ART clients have emerged, in part, because client groups do not require the same subset of services [64]. Clinics in our study incorporated differentiation into their OSS models by separating new PrEP initiators from those coming for PrEP refills. By building workflows around the different types of clients and their sets of needs, the OSS model created greater predictability in service times. Whereas high variation in service times often lowers the acceptability of public facility‐based services [65, 66, 67], the PrEP clients in our study expressed strong acceptance of a PrEP delivery model that featured shorter, more consistent wait times. Importantly, clinics reduced variation in service time without any additional human resources, making the OSS model a promising option for PrEP programs working within limited fiscal space.

Overall, the OSS model achieved its intended objectives, which were to improve efficiency of service delivery and care acceptability. As expected, we did not observe a significant change in provider contact time, initiations, or continuations. The OSS intervention that we tested did not entail any changes to the number or content of PrEP component services; however, it is possible that some portion of contact time is non‐value‐add, and future research should assess whether and how contact time could be reduced without compromising care quality. Our OSS intervention also did not entail a demand creation component (e.g., study clinics did not advertise OSS services). As such, we were not surprised to see no significant change in initiations; however, we recognize that increasing the number of clients with HIV risk who initiate PrEP will be important for maximizing PrEP's public health impact on population‐level HIV incidence. Additional research is needed to understand whether and how the OSS's gains in care acceptability (e.g., greater privacy) can be parlayed into more individuals initiating PrEP. Lastly, as we expected for a study of our size and duration, we did not observe a significant change in PrEP continuation. Additional research is needed to understand whether an OSS model affects PrEP continuation in the long term.

Our study has limitations. We interviewed English‐speaking providers and clients willing to deliver or obtain OSS PrEP services at a public clinic in western Kenya; their perspectives may not generalize to other providers/clients and PrEP clinics in other provinces of Kenya. We did not collect quantitative data on clinics’ fidelity to their OSS model. Future research should capture this information to understand at what level of fidelity the model needs to be executed to achieve the same outcomes. Most clients obtained follow‐up care at the OSS only once; it is possible that our post‐intervention period was not long enough to capture a lagged effect on continuation.

## 5 CONCLUSIONS

For PrEP to succeed as a public health intervention, it not only needs to be available at scale, but also used by the target population with sufficient rates of uptake, persistence and adherence [68]. An OSS approach to PrEP delivery may be useful for obtaining provider buy‐in and making care more patient‐centred.

## COMPETING INTERESTS

JMB is an employee of Gilead Sciences. For the remaining authors, none were declared.

## AUTHORS’ CONTRIBUTIONS

Study conceptualization and funding acquisition: KKM, JMB, EI, EAB and GO. Data collection tool development: SDR, JFM, GO, JO, SP, BK and AD. Data collection: BN, BK and AD. Project administration: SDR, JO, BN, SP and JFM. Data analysis: SDR, KKM, BK and AD. Writing – original draft: SDR. Writing – review and editing: All authors.

## FUNDING

The Partners Scale‐Up Project is funded by the National Institute of Mental Health of the US National Institutes of Health (grant R01 MH095507) and the Bill & Melinda Gates Foundation (grant OPP1056051).

## Supporting information


**Table S1**. Study clinics' PrEP delivery models pre‐ and post‐implementation of the One‐Stop Shop (OSS) intervention
**Table S2**. Consolidated criteria for reporting qualitative studies (COREQ) checklist
**Table S3**. Demographic characteristics of interview participants

## Data Availability

Data are available by contacting the International Clinical Research Center at the University of Washington (icrc@uw.edu).

## References

[1] World Health Organization . WHO expands recommendation on oral pre‐exposure prophlaxis of HIV infection (PrEP). Geneva: World Health Organization; 2015.

[2] PrEPWatch . Global PrEP Tracker. PrEP Watch; 2020 [cited 2020 Apr 13]. Available from: https://www.prepwatch.org/in‐practice/global‐prep‐tracker/

[3] Bavinton BR , Grulich AE . HIV pre‐exposure prophylaxis: scaling up for impact now and in the future. Lancet Public Health. 2021;6(7):e528–33. 34087117 10.1016/S2468-2667(21)00112-2

[4] Malley GO , Barnabee G , Mugwanya K . Scaling‐up PrEP delivery in sub‐Saharan Africa: what can we learn from the scale‐up of ART? Curr HIV/AIDS Rep. 2019;16:141–50. 30796608 10.1007/s11904-019-00437-6 PMC6469867

[5] Irungu EM , Baeten JM . PrEP rollout in Africa: status and opportunity. Nat Med. 2020;26(5):655–64. 32405065 10.1038/s41591-020-0872-x

[6] AVAC, & PATH, IAS . Bringing PrEP closer to home: why is now the time for differentiated PrEP? AIDS. 2020 [accessed 15 Sept 2020]. Available from: http://differentiatedservicedelivery.org/Resources/differentiated_PrEP_slides

[7] Were D , Musau A , Mutegi J , Ongwen P , Manguro G , Kamau M , et al. Using a HIV prevention cascade for identifying missed opportunities in PrEP delivery in Kenya: results from a programmatic surveillance study. J Int AIDS Soc. 2020;23(S3):67–77. 10.1002/jia2.25537 PMC7325512 32602658

[8] Ongolly F , Ngure K , Dolla A , Awour M , Irungu E , Mugo N , et al. Experiences of accessing PrEP in public HIV clinics: a case of Kenyan HIV‐uninfected people in serodiscordant relationships. AIDS Res Hum Retroviruses. 2018;34(S1):93.

[9] Patel RC , Stanford‐Moore G , Odoyo J , Pyra M , Wakhungu I , Anand K , et al. “Since both of us are using antiretrovirals, we have been supportive to each other”: facilitators and barriers of pre‐exposure prophylaxis use in heterosexual HIV serodiscordant couples in Kisumu, Kenya. J Int AIDS Soc. 2016;19(1):21134. 27964776 10.7448/IAS.19.1.21134 PMC5155127

[10] Camlin CS , Koss CA , Getahun M , Owino L , Itiakorit H , Akatukwasa C , et al. Understanding demand for PrEP and early experiences of PrEP use among young adults in rural Kenya and Uganda: a qualitative study. AIDS Behav. 2020. 24(7):2149–2162. 10.1007/s10461-020-02780-x 31955361 PMC7909847

[11] Pilgrim N , Mathur S , Gottert A , Rutenberg N , Pulerwitz J . Building evidence to guide PrEP introduction for adolescent girls and young women. Washington, DC: Population Council; 2016. 10.1016/S2352-3018(16)30115-1 27562739

[12] Mayer KH , Allan‐Blitz LT . PrEP 1.0 and beyond: optimizing a biobehavioral intervention. J Acquir Immune Defic Syndr. 2019;82:S113–7. 31658197 10.1097/QAI.0000000000002169 PMC6830954

[13] Scully EP , Weld ED , Blankson JN . Challenges in optimizing preexposure prophylaxis development, engagement, and access for HIV prevention. J Clin Invest. 2019;129(12):5071–3. 31710315 10.1172/JCI134389 PMC6877325

[14] Aaron E , Blum C , Seidman D , Hoyt MJ , Simone J , Sullivan M , et al. Optimizing delivery of HIV preexposure prophylaxis for women in the United States. AIDS Patient Care STDs. 2018;32(1):16–23. 29323558 10.1089/apc.2017.0201 PMC5756936

[15] Landovitz RJ . Optimizing delivery of preexposure prophylaxis—the next frontier. JAMA Intern Med. 2016;176(1):85–6. 26571121 10.1001/jamainternmed.2015.6530

[16] Kenya Ministry of Health, National AIDS Control Council . Kenya AIDS strategic framework II 2020/21–2024/2025. Nairobi: Ministry of Health; 2021.

[17] WHO . Contnuity and coordinaton of care: a practce brief to support implementaton of the WHO Framework on integrated people‐centred health services. Geneva: WHO; 2018.

[18] French R , Coope C , Graham A , Gerressu MCS , Stephenson J . One stop shop versus collaborative integration: what is the best way of delivering sexual health services ? Sex Transm Infect. 2006;82(3):202–6. 16731668 10.1136/sti.2005.018093 PMC2564738

[19] Owiti P , Zachariah R , Bissell K , Kumar AMV , Diero L , Carter EJ , et al. Integrating tuberculosis and HIV services in rural Kenya: uptake and outcomes. Public Health Action. 2015;5(1):36–44. 26400600 10.5588/pha.14.0092 PMC4525370

[20] Management Sciences for Health . One‐stop shop for TB/HIV services: a model for increasing antiretroviral therapy uptake in Uganda. 2017 [accessed 10 Mar 2021]. Available from: https://www.msh.org/resources/one‐stop‐shop‐for‐tbhiv‐services‐a‐model‐for‐increasing‐antiretroviral‐therapy‐uptake‐in

[21] Herce ME , Morse J , Luhanga D , Harris J , Smith HJ , Besa S , et al. Integrating HIV care and treatment into tuberculosis clinics in Lusaka, Zambia: results from a before‐after quasi‐experimental study. BMC Infect Dis. 2018;18(1):536. 30367622 10.1186/s12879-018-3392-2 PMC6204013

[22] Ansa GA , Walley JD , Siddiqi K , Wei X . Assessing the impact of TB/HIV services integration on TB treatment outcomes and their relevance in TB/HIV monitoring in Ghana. Infect Dis Poverty. 2012;1(1):1–8. 23849044 10.1186/2049-9957-1-13 PMC3710204

[23] Grossman D , Onono M , Newmann SJ , Blat C , Bukusi EA , Shade SB , et al. Integration of family planning services into HIV care and treatment in Kenya : a cluster‐randomized trial. AIDS. 2013;27(Suppl 1):S77–85. 10.1097/QAD.0000000000000035 24088687

[24] Cohen CR , Grossman D , Onono M , Blat C , Newmann J , Burger RL , et al. Integration of family planning services into HIV care clinics: results one year after a cluster randomized controlled trial in Kenya. PLoS One. 2017;12:e0172992. 28328966 10.1371/journal.pone.0172992 PMC5362197

[25] Church K , Wringe A , Lewin S , Ploubidis GB , Fakudze P , Initiative I , et al. Exploring the feasibility of service integration in a low‐income setting: a mixed methods investigation into different models of reproductive health and HIV care in Swaziland. PLoS One. 2015;10(5):e0126144. 25978632 10.1371/journal.pone.0126144 PMC4433110

[26] Hoke T , Harries J , Crede S , Green M , Constant D , Petruney T , et al. Expanding contraceptive options for PMTCT clients: a mixed methods implementation study in Cape Town, South Africa. Reprod Health. 2014;11(1):3. 24410922 10.1186/1742-4755-11-3 PMC3895666

[27] Phiri S , Feldacker C , Chaweza T , Mlundira L , Tweya H , Speight C , et al. Integrating reproductive health services into HIV care: strategies for successful implementation in a low‐resource HIV clinic in Lilongwe, Malawi. J Fam Plan Reprod Health Care. 2016;42(1):17–23. 10.1136/jfprhc-2013-100816 PMC4717379 25902815

[28] Sarnquist CC , Moyo P , Stranix‐Chibanda L , Chipato T , Kang JL , Maldonado YA . Integrating family planning and prevention of mother to child HIV transmission in Zimbabwe. Contraception. 2014;89(3):209–14. 24332254 10.1016/j.contraception.2013.11.003 PMC3965605

[29] Irungu E , Mugwanya K , Mugo N , Bukudi E , Donnell D , Odoyo J , et al. Integrating oral pre‐exposure prophylaxis services to public HIV care clinics in Kenya: results from a pragmatic stepped‐wedge randomized trial [abstract]. J Int AIDS Soc. 2021;24(S1):PE16.18.

[30] Shea CM , Jacobs SR , Esserman DA , Bruce K , Weiner BJ . Organizational readiness for implementing change: a psychometric assessment of a new measure. Implement Sci. 2014;9(1):7. 24410955 10.1186/1748-5908-9-7 PMC3904699

[31] Creswell JW , Plano Clark VL . Core mixed methods designs. In: Designing and conducting mixed methods research. 3rd ed. Thousand Oaks, CA: Sage Publications, Inc.; 2018, p. 51–100.

[32] Palinkas LA , Aarons GA , Horwitz S , Chamberlain P , Hurlburt M , Landsverk J . Mixed method designs in implementation research. Adm Policy Ment Health. 2011;38(1):44–53. 20967495 10.1007/s10488-010-0314-z PMC3025112

[33] Hsieh H‐F , Shannon SE . Three approaches to qualitative content analysis. Qual Health Res. 2005;15(9):1277–88. 16204405 10.1177/1049732305276687

[34] Powell BJ , Waltz TJ , Chinman MJ , Damschroder LJ , Smith JL , Matthieu MM , et al. A refined compilation of implementation strategies: results from the Expert Recommendations for Implementing Change (ERIC) project. Implement Sci. 2015;10(1):21. 25889199 10.1186/s13012-015-0209-1 PMC4328074

[35] Perry CK , Damschroder LJ , Hemler JR , Woodson TT , Ono SS , Cohen DJ . Specifying and comparing implementation strategies across seven large implementation interventions: a practical application of theory. Implement Sci. 2019;14(1):1–13. 30898133 10.1186/s13012-019-0876-4 PMC6429753

[36] Ohno T . Toyota production system: beyond large‐scale production. Boca Raton, FL: Taylor & Francis Group; 1988.

[37] Langley G , Moen R , Nolan K , Nolan T , Norman C , Provost L . The improvement guide. 2nd ed. San Francisco, CA: Jossey‐Bass; 2009.

[38] Saldaña J . The coding manual for qualitative researchers. 2nd ed. London: Sage Publications, Ltd.; 2013.

[39] Miles MB , Huberman MA , Saldana J . Fundamentals of qualitative data analysis. In: Qualitative data analysis: a method sourcebook. 3rd ed. Thousand Oaks, CA: Sage Publications, Inc.; 2014, p. 69–75.

[40] Attride‐Stirling J . Thematic networks: an analytic tool for qualitative research. Qual Res. 2001;1(3):385–405.

[41] Morse JM . Simultaneous and sequential qualitative mixed method designs. Qual Inq. 2010;16(6):483–91.

[42] Creswell JW , Plano Clark VL . Analyzing and interpreting data in mixed methods research. In: Designing and conducting mixed methods research. 3rd ed. Thousand Oaks, CA: Sage Publications, Inc.; 2018, p. 209–58.

[43] Flottorp SA , Oxman AD , Krause J , Musila NR , Wensing M , Godycki‐Cwirko M , et al. A checklist for identifying determinants of practice: a systematic review and synthesis of frameworks and taxonomies of factors that prevent or enable improvements in healthcare professional practice. Implement Sci. 2013;8(35). 10.1186/1748-5908-8-35 PMC3617095 23522377

[44] Squires JE , Aloisio LD , Grimshaw JM , Bashir K , Dorrance K , Coughlin M , et al. Attributes of context relevant to healthcare professionals’ use of research evidence in clinical practice: a multi‐study analysis. Implement Sci. 2019;14(1):1–14. 31113449 10.1186/s13012-019-0900-8 PMC6530177

[45] Saul J , Bachman G , Allen S , Toiv NF , Cooney C , Beamon TA . The DREAMS core package of interventions: a comprehensive approach to preventing HIV among adolescent girls and young women. PLoS One. 2018;13(12):e0208167. 30532210 10.1371/journal.pone.0208167 PMC6285267

[46] Ortblad KF . Pharmacy delivery to expand the reach of PrEP in Kenya. 2020 [cited 2021 Mar 15]. Available from: https://clinicaltrials.gov/ct2/show/NCT04558554 10.1002/jia2.25619 PMC7525802 32996721

[47] Mccollum R , Theobald S , Otiso L , Martineau T , Karuga R , Barasa E , et al. Priority setting for health in the context of devolution in Kenya: implications for health equity and community‐based primary care. Health Policy Plan. 2018;33(6);729–42 10.1093/heapol/czy043 29846599 PMC6005116

[48] Mack AN , Wong C , Mckenna K , Lemons A , Odhiambo J , Agot K . Human resource challenges to integrating HIV pre‐exposure prophylaxis (PrEP) into the public health system in Kenya: a qualitative study. Afr J Reprod Health. 2015;19(1):54–62. 26103695

[49] Ahmed N , Pike C , Bekker LG . Scaling up pre‐exposure prophylaxis in sub‐Saharan Africa. Curr Opin Infect Dis. 2019;32(1):24–30. 30461452 10.1097/QCO.0000000000000511

[50] Jackson‐Gibson M , Ezema A , Orer W , Were I , Ohiomoba ROO , Mbullo PO . Facilitators and barriers to pre‐exposure prophylaxis (PrEP) uptake among adolescent girls and young women in Seme‐Sub County, Kisumu, Kenya. BMC Public Health. 2021;21:1284. 34210288 10.1186/s12889-021-11335-1 PMC8252310

[51] Stankevitz K , Nhamo D , Murungu J , Ridgeway K , Mamvuto T , Lenzi R , et al. Test and prevent: evaluation of a pilot program linking clients with negative HIV test results to pre‐exposure prophylaxis in Zimbabwe. Glob Health Sci Pract. 2021;9(1):40–54. 33795361 10.9745/GHSP-D-20-00444 PMC8087428

[52] Lanham M , Ridgeway K , Mireku M , Stankevitz K , Kyongo J . Health care providers’ attitudes toward and experiences delivering oral PrEP to adolescent girls and young women in Kenya, South Africa, and Zimbabwe. BMC Health Serv Res. 2021;21:1112. 10.1186/s12913-021-06978-0 34663320 PMC8522219

[53] Herzberg F , Mausner B , Snyderman B . The motivation to work. Hoboken, NJ: John Wiley & Sons, Inc.; 1959.

[54] Damschroder LJ , Aron DC , Keith RE , Kirsh SR , Alexander JA , Lowery JC . Fostering implementation of health services research findings into practice: a consolidated framework for advancing implementation science. Implement Sci. 2009;4(1):50. 19664226 10.1186/1748-5908-4-50 PMC2736161

[55] Ciapponi A , Lewin S , Herrera CA , Opiyo N , Pantoja T , Paulsen E , et al. Delivery arrangements for health systems in low‐income countries: an overview of systematic reviews. Cochrane Database Syst Rev. 2017;53(9):1689–99. 10.1002/14651858.CD011083.pub2 PMC5621087 28901005

[56] Crowley T , Mayers P . Trends in task shifting in HIV treatment in Africa: effectiveness, challenges and acceptability to the health professions. Afr J Prim Health Care Fam Med. 2015;7(1):807. 10.4102/phcfm.v7i1.807 PMC4564830 26245622

[57] Magidson JF , Grouse H , Psaros C , Remmert JE , O'Cleirigh C , Safren SA . Task shifting and delivery of behavioral medicine interventions in resource‐poor global settings: HIV/AIDS treatment in sub‐Saharan Africa. In: Vranceanu A‐M , GreerSteven JA , Safren SA , editors. The Massachusetts General Hospital handbook of behavioral medicine: a clinician's guide to evidence‐based psychosocial interventions for individuals with medical illness. New York: Humana Press; 2016, p. 297–320.

[58] Charns MP , Smith Tewksbury LJ . The Continuum of Organization Structures. Collaborative Management in Health Care: Implementing the Integrative Organization. 1st ed, San Francisco: Jossey‐Bass Publishers; 1993;20–65.

[59] Dlamini‐Simelane T , Moyer E . Task shifting or shifting care practices? The impact of task shifting on patients’ experiences and health care arrangements in Swaziland. BMC Health Serv Res. 2017;17(1):1–12. 28069047 10.1186/s12913-016-1960-y PMC5223454

[60] Church K , Wringe A , Fakudze P , Kikuvi J , Simelane D , Mayhew SH . The relationship between service integration and client satisfaction: a mixed methods case study within HIV services in a high prevalence setting in Africa. AIDS Patient Care STDs. 2012;26(11):662–73. 23078548 10.1089/apc.2012.0191

[61] Nhassengo P , Cataldo F , Magac A , Hoffman RM , Nerua L , Saide M , et al. Barriers and facilitators to the uptake of test and treat in Mozambique: a qualitative study on patient and provider perceptions. PLoS One. 2018;13(12):e0205919. 30586354 10.1371/journal.pone.0205919 PMC6306230

[62] Wanga V , Omollo V , Bukusi E , Odoyo J , Morton J , Kidoguchi L , et al. Uptake and impact of facility‐based HIV self‐testing on PrEP delivery: a pilot study among young women in Kisumu, Kenya. J Int AIDS Soc. 2020;23(8):e25561. 32820597 10.1002/jia2.25561 PMC7441009

[63] Ortblad KF , Kearney JE , Mugwanya K , Irungu EM , Haberer JE , Barnabas RV , et al. HIV‐1 self‐testing to improve the efficiency of pre‐exposure prophylaxis delivery: a randomized trial in Kenya. Trials. 2019;20(1):396. 31272495 10.1186/s13063-019-3521-2 PMC6610957

[64] International AIDS Society . Stable client models. 2021 [cited 2021 Feb 25]. Available from: https://differentiatedservicedelivery.org/Models/Treatment

[65] Campbell C , Scott K , Madanhire C , Nyamukapa C , Gregson S . A “good hospital”: nurse and patient perceptions of good clinical care for HIV‐positive people on antiretroviral treatment in rural Zimbabwe—a mixed‐methods qualitative study. Int J Nurs Stud. 2011;48(2):175–83. 20801450 10.1016/j.ijnurstu.2010.07.019 PMC3037471

[66] Ware NC , Wyatt MA , Geng EH , Kaaya SF , Agbaji OO , Muyindike WR , et al. Toward an understanding of disengagement from HIV treatment and care in sub‐Saharan Africa: a qualitative study. PLoS Med. 2013;10(1):e1001369. 23341753 10.1371/journal.pmed.1001369 PMC3541407

[67] Hardon AP , Akurut D , Comoro C , Ekezie C , Irunde HF , Gerrits T , et al. Hunger, waiting time and transport costs: time to confront challenges to ART adherence in Africa. AIDS Care. 2007;19(5):658–65. 17505927 10.1080/09540120701244943

[68] Pyra MN , Haberer JE , Hasen N , Reed J , Mugo NR , Baeten JM . Global implementation of PrEP for HIV prevention: setting expectations for impact. J Int AIDS Soc. 2019;22(8):e25370. 31456348 10.1002/jia2.25370 PMC6712462

