# Supplementary material for: A one‐stop shop model for improved efficiency of pre‐exposure prophylaxis delivery in public clinics in western Kenya: a mixed methods implementation science study
Source: J Int AIDS Soc. 2021 Dec 12;24(12):e25845. doi: 10.1002/jia2.25845 (PMC8666585; doi:10.1002/jia2.25845)
Supplement: Supplementary file 1 — Table S1. Study clinics' PrEP delivery models pre‐ and post‐implementation of the One‐Stop Shop (OSS) intervention Table S2. Consolidated criteria for reporting qualitative studies (COREQ) checklist Table S3. Demographic characteristics of interview participants [file JIA2-24-e25845-s001.docx]

# Appendix

Below, we provide additional details about study clinics’ pre-intervention PrEP delivery models and OSS models in **Table S1**, our qualitative methodology in **Table S2,** and interview participant demographics in **Table S3**.

| **Table S1**. Study clinics’ PrEP delivery models pre- and post-implementation of the One-Stop Shop (OSS) intervention | | | | | | | | | | | | | | |
| --- | --- | --- | --- | --- | --- | --- | --- | --- | --- | --- | --- | --- | --- | --- |
|  | **Where (*and who*) performed each PrEP component service or delivery task** | | | | | | | | | | | | | |
| **PrEP component service or delivery task^†^** | **Clinic A** | | | **Clinic B** | | | | | **Clinic C** | | | | **Clinic D** | |
|  | **Pre**  **(CCC)** | **Post**  **(DFC)** | | **Pre**  **(CCC)** | | **Post**  **(DFC)** | | | **Pre**  **(CCC)** | | **Post**  **(FP)** | | **Pre**  **(CCC)** | **Post**  **(GBV)** |
| Client file retrieval | CCC adherence room  *(adherence counselor or peer educator)* | *OSS*  *(OSS clinician or peer educator)* | | CCC reception desk  *(client obtains file from records clerk)* | | OSS  *(OSS clinician or peer educator)* | | | CCC clinical room  *(CCC clinician)* | | OSS  *(OSS clinician)* | | CCC adherence room  *(client obtains file from adherence counselor)* | OSS  *(OSS clinician)* |
| Vital signs assessment | *CCC triage area*  *(peer educator)* | *OSS*  *(OSS clinician or peer educator)* | | CCC triage area  *(peer educator or adherence counselor)* | | OSS  *(OSS clinician or peer educator)* | | | CCC triage area  *(peer educator)* | | FP clinic triage area  *(OSS clinician)* | | CCC triage area  *(peer educator)* | OSS  *(OSS clinician)* |
| HIV testing^‡^ and risk assessment | HTS point in adjacent building  *(HTS counselor)* | First three months of post-intervention period: ^§^  HTS point in same building as OSS  *(HTS counselor)*  Remainder of post-intervention period:  HTS point in separate building as OSS  *(HTS counselor)* | | CCC HTS point  *(HTS counselor)* | | First three months of post-intervention period: ^§^  HTS point in same building as OSS  *(HTS counselor)*  Remainder of post-intervention period:  HTS point in separate building as OSS  *(HTS counselor)* | | | CCC HTS point  *(HTS counselor)* | | HTS point in same building as OSS  *(HTS counselor)* | | CCC HTS point  *(HTS counselor)* | Original plan: OSS  *(HTS provider)*  As implemented: HTS point in separate building as OSS  *(HTS provider)* |
| Risk reduction counseling | CCC clinical room  *(CCC clinician, peer educator, or adherence counselor)* | OSS  *(OSS clinician or peer educator)* | | CCC clinical room  *(CCC clinician)* | | OSS  *(OSS clinician)* | | | CCC clinical room  *(CCC clinician)* | | OSS  *(OSS clinician)* | | CCC clinical room  *(CCC clinician or adherence counselor)* | OSS  *(OSS clinician)* |
| Clinical review^¶^ and prescription writing | CCC clinical room  *(CCC clinician)* | OSS  *(OSS clinician)* | | CCC clinical room  *(CCC clinician)* | | OSS  *(OSS clinician)* | | | CCC clinical room  *(CCC clinician)* | | OSS  *(OSS clinician)* | | CCC clinical room  *(CCC clinician)* | OSS  *(OSS clinician)* |
| Adherence counseling | CCC clinical room  *(CCC clinician, adherence counselor, or peer educator)* | OSS  *(OSS clinician or peer educator)* | | CCC clinical room  *(CCC clinician or adherence counselor)* | | OSS  *(OSS clinician)* | | | CCC clinical room  *(CCC clinician)* | | OSS  *(OSS clinician)* | | CCC adherence room or CCC clinical room  *(adherence counselor or CCC clinician)* | OSS  *(OSS clinician)* |
| PrEP dispensing | CCC pharmacy  *(pharmacy provider)* | OSS  *(OSS clinician or peer educator)* | | CCC pharmacy *(pharmacy provider)* | | OSS  (*OSS clinician or peer educator)* | | | CCC pharmacy *(pharmacy provider)* | | OSS  (*OSS clinician)* | | CCC pharmacy *(pharmacy provider)* | OSS  (*OSS clinician)* |
| Booking next appointment | CCC clinical room  *(CCC clinician or peer educator)* | OSS  *(CCC clinician or peer educator)* | | CCC reception desk  *(records clerk)* | | OSS  *(OSS clinician or peer educator)* | | | CCC clinical room  *(CCC clinician)* | | OSS  *(OSS clinician)* | | CCC booking area  *(CCC clinician or peer educator)* | OSS  *(OSS clinician)* |
| Client file storage | CCC adherence room  *(peer educator)* | OSS  *(OSS clinician or peer educator)* | | CCC reception desk  *(CCC clinician returns file to records clerk for storage)* | | OSS  *(OSS clinician or peer educator)* | | | CCC clinical room  *(CCC clinician)* | | OSS  *(OSS clinician)* | | CCC adherence room  *(peer educator)* | OSS  *(OSS clinician)* |
| Total number of providers seen | 4 to 5 | 2 to 3 | | 5 to 6 | | 2 to 3 | | | 4 | | 2 | | 4 to 5 | 2 |
| Total number of different clinic areas visited | 5 | 3 | | 5 | | 2 | | | 4 | | 3 | | 6 | Original plan: 1  As implemented: 2 |
| ^†^Delivery tasks are steps of the PrEP delivery process, such as client file retrieval, that are not core components of the PrEP intervention but have to occur for the client to receive PrEP services at the clinic. Some PrEP component services, such as HTS, are divvied into several component services to capture more granular detail about client movement to receive these services in different areas of the clinic. ^‡^In line with national guidelines, clients with good adherence receive quarterly HIV testing after three months of PrEP use. In some cases, new initiators receive HIV testing at an HTS point in a different department (e.g., the outpatient department) and are referred to the CCC or OSS for PrEP. HTS counselors generally conduct a brief HIV risk assessment, and the clinician performs more in-depth HIV risk reduction counseling. ^§^At clinics A and B, the HTS point located in the same building as the OSS was temporarily closed as a COVID-19 precaution about four months into the post-intervention period; thereafter, OSS clients received HIV testing at an HTS point in a separate building. ^¶^The primary purpose of the clinical review component is to ensure that the client is (or continues to be) medically eligible for PrEP. After initiating PrEP, clients undergo clinical review approximately every three months. Its content varies depending on client type (new initiator or follow-up client) but generally involves taking client medical history, performing physical exam, and assessing PrEP side effects. Unless experiencing issues that require clinician attention (e.g., PrEP side effects), follow-up clients not due for their quarterly HIV testing and clinical review (i.e., “refill-only clients”) skip this step. Abbreviations: CCC = HIV comprehensive care clinic; DFC = Differentiated care clinic; FP = Family planning clinic; GBV = Gender-based violence clinic; PrEP = pre-exposure prophylaxis | | | | | | | | | | | | | | |
|  | | |  | |  | |  |  | |  | |  | | |

| **Table S2**. Consolidated criteria for reporting qualitative studies (COREQ) checklist | | |
| --- | --- | --- |
| **Domain** | **No. Item** | **Guide Questions/Description** |
| Research Team and Reflexivity | ***Personal Characteristics*** | |
|  | 1. Interviewer/facilitator | Coauthors BK and AD, both Kenyan qualitative researchers, conducted all interviews. |
|  | 1. Credentials | SDR has a Master’s in Public Health and a BA in Cultural Anthropology. BK and AD have extensive experience conducting qualitative interviews for HIV prevention research studies. |
|  | 1. Occupation | SDR is a research analyst and PhD candidate in Global Health Implementation Science at University of Washington. BK and AD are qualitative research assistants for the Kenya Medical Research Institute. |
|  | 1. Gender | SDR and AD are female, and BK is male. |
|  | 1. Experience and training | SDR has completed doctoral-level courses in qualitative research. BK and AD have extensive experience collecting and analyzing qualitative data and have authored or coauthored numerous articles and abstracts for qualitative studies. |
|  | ***Relationship with Participants*** | |
|  | 1. Relationship established | Relationships were limited to interviews. |
|  | 1. Participant knowledge of interviewer | Prior to beginning the interviews, BK and AD provided participants with a general description of the study (e.g., its objectives and details of participation) and a brief personal introduction. |
|  | 1. Interviewer characteristics | We do not report additional interviewer characteristics. |
| Study Design | ***Theoretical Framework*** | |
|  | 1. Methodological orientation and theory | Our study draws on approaches from both conventional and directed content analysis. We created de novo semi-structured interview guides of open-ended questions. During analysis, we derived our coding categories directly from the text data, from Ohno’s model for continuous quality improvement, and from Langley et al.’s compilation of change concepts. (Citations are included in the reference list.) |
|  | ***Participant Selection*** | |
|  | 1. Sampling | We used purposive sampling to recruit healthcare providers employed by OSS clinics or the county department of health and PrEP clients. We intentionally sought out providers from a variety of professional cadres (e.g., clinical officers, nurses, peer educators) and clients of different sexes, ages, and prior experience with the clinic’s pre-OSS PrEP delivery model. |
|  | 1. Method of approach | Interviewers approached potential client participants as they exited the OSS. With TA assistance, interviewers identified eligible providers and approached them in person or by phone. |
|  | 1. Sample size | 29 interviews completed (14 with providers and 15 with clients) |
|  | 1. Non-participation | All invited providers agreed to participate. We did not track the number of PrEP clients who were approached and declined to participate in an interview. |
|  | ***Setting*** | |
|  | 1. Setting of data collection | All interviews took place in a private room at the study clinic or via phone. |
|  | 1. Presence of non-participants | Aside from the interviewer and the participant, no other individuals were present during the interviews. |
|  | 1. Description of sample | Adult PrEP clients and adult PrEP providers and/or clinic administrators. |
|  | ***Data Collection*** | |
|  | 1. Interview guide | We developed and pilot tested two de novo semi-structured interview guides (one for providers and one for clients). |
|  | 1. Repeat interviews | We interviewed each participant once (i.e., no repeat interviews). |
|  | 1. Audio/visual recording | All interviews were audio-recorded with participant consent. |
|  | 1. Field notes | We did not collect field notes. |
|  | 1. Duration | Interviews lasted approximately one hour. |
|  | 1. Data saturation | We did not conduct interviews to the point of data saturation; however, given the tightly scoped nature of our research question, our analytic goals (to discern themes, rather than develop theory), and the relative homogeneity of our sample (all OSS providers and all OSS PrEP clients), we anticipated reaching code saturation after 12 interviews per group. We, therefore, aimed to interview 3 providers and 3 clients from each OSS clinic. |
|  | 1. Transcripts returned | Transcripts were not returned to participants for comment or correction. |
| Analysis and Findings | ***Data Analysis*** | |
|  | 1. Number of data coders | One (SDR) |
|  | 1. Description of the coding tree | SDR drafted interview memos that included a summary of key points for each code and quotations. Memos were reviewed by BK and AD, with disagreements resolved through group discussion. |
|  | 1. Derivation of themes | In each interview memo, SDR included analytic reflections that drew comparisons across participants and datasets and synthesized findings into higher-level themes. |
|  | 1. Software | NVivo 12 Pro |
|  | 1. Participant checking | Participants did not provide feedback on our findings. |
|  | ***Reporting*** | |
|  | 1. Quotations presented | Illustrative quotes are presented in the body of the paper as well as Tables 2 and 3. |
|  | 1. Data and findings consistent | Yes |
|  | 1. Clarity of major themes | Major themes are presented in the results section under the sub-sections “OSS impact on service time”, “implementation challenges”, and “OSS impact on service quality”. We further discuss the relevance of these themes in the discussion section. |
|  | 1. Clarity of minor themes | Not applicable. (We do not present minor themes.) |

| **Table S3.** Demographic characteristics of interview participants | | |
| --- | --- | --- |
| **Characteristic** | **PrEP Providers**  (N=14) | **PrEP Clients**  (N=15) |
| Female sex – no. (%) | 6 (43) | 8 (53) |
| Age – median (IQR) | 34 (33-38) | 39 (29-41) |
| Educational attainment – no (%)^†^ |  |  |
| Less than high school graduate | 0 (0) | 5 (33) |
| High school graduate | 0 (0) | 5 (33) |
| Some college or college certificate/diploma | 10 (71) | 3 (20) |
| Some university or university degree | 4 (29) | 2 (13) |
| Occupation – no. (%) |  |  |
| Clinical officer | 5 (36) | 0 (0) |
| Nurse | 4 (29) | 0 (0) |
| Healthcare counselor^‡^ | 2 (14) | 0 (0) |
| Healthcare administrator | 3 (21) | 0 (0) |
| Business/salesperson | 0 (0) | 4 (27) |
| Farmer | 0 (0) | 3 (20) |
| Hospitality/service industry worker | 0 (0) | 2 (13) |
| Other^§^ | 0 (0) | 6 (40) |
| Married – no. (%) | - | 13 (87) |
| Number of children – median (IQR) | - | 3 (2-5) |
| Monthly household income in Kenyan shillings^¶^ – median (IQR) | - | 30,000 (19,000-47,500) |
| Lives in rural area^††^ – no. (%) | - | 7 (47) |
| ^†^In the Kenyan higher education system, college certificates, college diplomas, and university degrees generally take a minimum of 1, 2, and 4 years, respectively, to complete; ^‡^Includes psychologist, adherence counselor, identification and retention assistant, and peer educator; ^§^Includes, for example, pastor, homemaker, caregiver, and clerk; ^¶^Approximately USD $270 ($170-430); ^††^Self-reported by participant | | |
